# Superconducting graphene sheets in CaC_6_ enabled by phonon-mediated interband interactions

**DOI:** 10.1038/ncomms4493

**Published:** 2014-03-20

**Authors:** S.-L. Yang, J. A. Sobota, C. A. Howard, C. J. Pickard, M. Hashimoto, D. H. Lu, S.-K. Mo, P. S. Kirchmann, Z.-X. Shen

**Affiliations:** 1 Stanford Institute for Materials and Energy Sciences, SLAC National Accelerator Laboratory, 2575 Sand Hill Road, Menlo Park, California 94025, USA; 2 Geballe Laboratory for Advanced Materials, Departments of Physics and Applied Physics, Stanford University, Stanford, California 94305, USA; 3 London Centre for Nanotechnology and Department of Physics and Astronomy, University College London, London WC1E 6BT, UK; 4 Stanford Synchrotron Radiation Lightsource, SLAC National Accelerator Laboratory, 2575 Sand Hill Road, Menlo Park, California 94025, USA; 5 Advanced Light Source, Lawrence Berkeley National Laboratory, Berkeley, California 94720, USA

## Abstract

There is a great deal of fundamental and practical interest in the possibility of inducing superconductivity in a monolayer of graphene. But while bulk graphite can be made to superconduct when certain metal atoms are intercalated between its graphene sheets, the same has not been achieved in a single layer. Moreover, there is a considerable debate about the precise mechanism of superconductivity in intercalated graphite. Here we report angle-resolved photoelectron spectroscopy measurements of the superconducting graphite intercalation compound CaC_6_ that distinctly resolve both its intercalant-derived interlayer band and its graphene-derived π* band. Our results indicate the opening of a superconducting gap in the π* band and reveal a substantial contribution to the total electron–phonon-coupling strength from the π*-interlayer interband interaction. Combined with theoretical predictions, these results provide a complete account for the superconducting mechanism in graphite intercalation compounds and lend support to the idea of realizing superconducting graphene by creating an adatom superlattice.

## Abstract (web summary)

Although superconductivity hasn't been observed in a sheet of graphene it is found in metal intercalated graphite. A high-resolution ARPES study of CaC_6_ conducted by Yang *et al.* provides strong clues as to the origin of superconductivity in these compounds and of ways to induce superconductivity in graphene.

## Graphical abstract

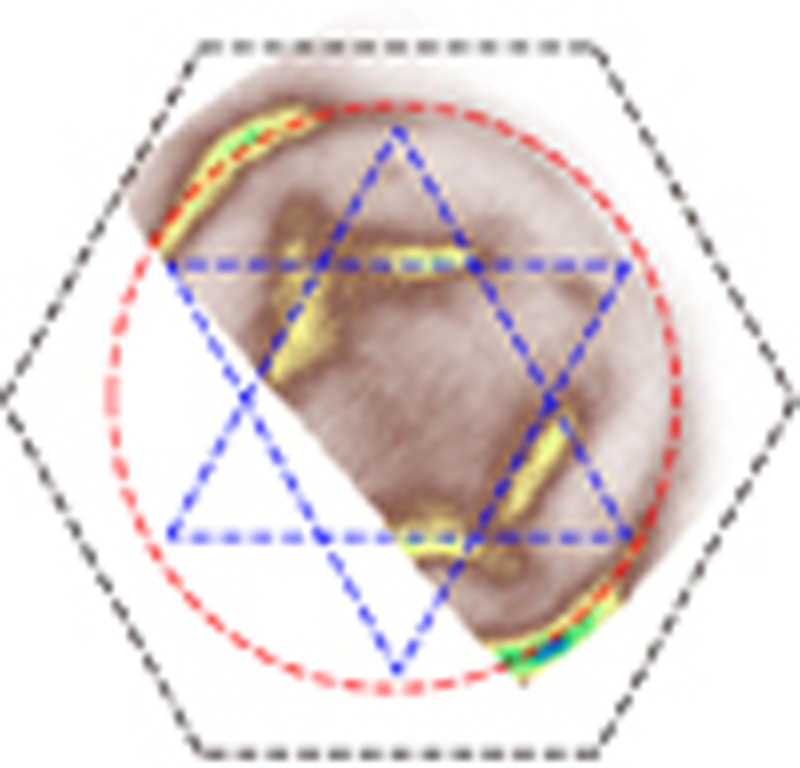

The debate on the superconducting mechanism of CaC_6_ has centred on the respective roles of the π* and interlayer (IL) bands^1^,^2^,^3^,^4^,^5^,^6^,^7^,^8^,^9^,^10^,^11^,^12^,^13^,^14^,^15^. Some theoretical studies proposed that strong phonon-mediated interactions between these two bands are required to explain superconductivity^1^,^4^,^5^,^6^, and would lead to both bands being superconducting and having superconducting gaps. This fully gapped picture was consistent with a number of experiments^7^,^8^,^9^,^10^, but challenged by angle-resolved photoelectron spectroscopy (ARPES)^11^,^12^,^13^. Valla *et al.*^11^ claimed that electron–phonon coupling (EPC) involving the π* band alone can explain superconductivity, and found no evidence for the existence of the IL band. In contrast, Sugawara *et al.*^12^ reported that the π* band is gapless, which suggested that superconductivity exists on the IL band only. Clearly, no consensus has been reached regarding which bands participate in superconductivity.

As we show in this Article, our ARPES measurement resolves the π* and IL bands with unprecedented clarity. Our analysis of the superconducting gaps and EPC on both bands yields compelling evidence for the picture in which the π*-IL interband interaction contributes >50% to the total EPC strength to enable superconductivity^1^,^4^,^5^,^6^.

## Results

### Electronic band structure

**Figure 1:**
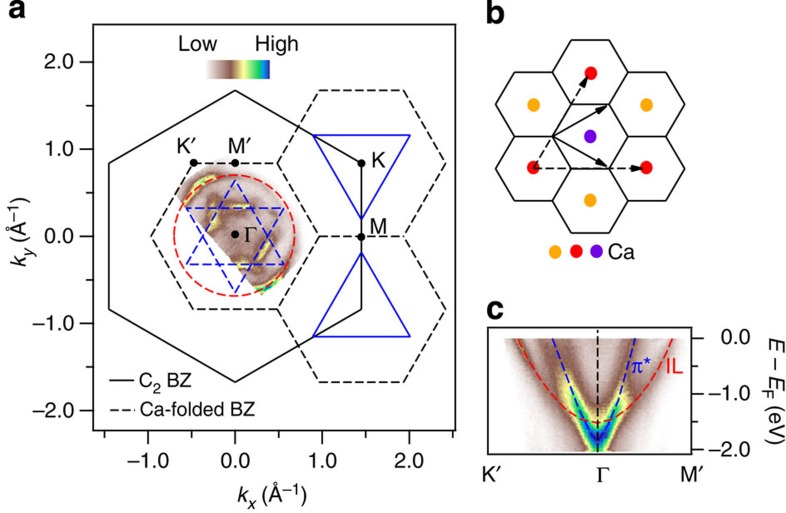
ARPES measurement revealing the CaC_6_ band structure near the zone centre. (**a**) Fermi surface map plotted in both the C_2_ (solid) Brillouin zone (BZ) and the Ca-folded (dashed) BZ. The solid blue triangles are schematic representations of the heavily electron-doped graphene π* pockets^11^. The dashed blue triangles illustrate the superlattice-folded π* pockets near Γ. The dashed red circle marks the IL pocket. (**b**) CaC_6_ crystal structure viewed along the *c*-axis. The black honeycomb lattice represents the graphene planes. The yellow, red and purple balls stand for the calcium atoms. Each colour corresponds to each calcium layer. The solid and dashed arrows are the two-dimensional lattice vectors of the graphene lattice and the calcium superlattice, respectively. (**c**) Electronic band dispersions along the K′-Γ-M′ trajectory. The blue- and red-guiding lines correspond to the π* and IL bands, respectively.

We begin by presenting the Fermi surface map taken with 9 eV photons in Fig. 1a. The most pronounced features are a warped hexagonal pocket and a near-circular pocket. The hexagonal pocket can be understood as a folding of the triangular graphene π* pockets^16^,^17^ owing to the calcium superlattice illustrated in Fig. 1b. At the same time, this band-folding cannot account for the near-circular pocket. Its near-circular shape, parabolic band dispersion (Fig. 1c) and three-dimensionality (Supplementary Fig. 1) all agree with the predicted free-electron-like character of the IL band^1^,^2^,^3^,^4^,^5^,^6^, and therefore permit its confident assignment. We note that the spectral feature identified as the IL band by Sugawara *et al.*^12^ spanned a region of momentum-space which contains the folded π* bands. Their inability to resolve these separate contributions undermined their claim of observing superconductivity associated with only the IL band. In contrast, the radius of our observed IL pocket is approximately twice as large as that measured by Sugawara *et al.*^12^, and is ∼50% larger than the calculated value at *k*_z_=0 using density functional theory^1^,^2^,^3^,^4^,^5^,^6^. While the former difference can be understood as our photon energy probing a *k*_z_ value closer to zero (Supplementary Fig. 1), the latter demonstrates that density functional theory underestimates the actual IL pocket size. Importantly, these differences allow us to clearly distinguish between the two Fermi surfaces near Γ, and therefore study the superconducting gaps and EPC on the individual bands to address the superconducting mechanism.

### Opening of superconducting gaps

**Figure 2:**
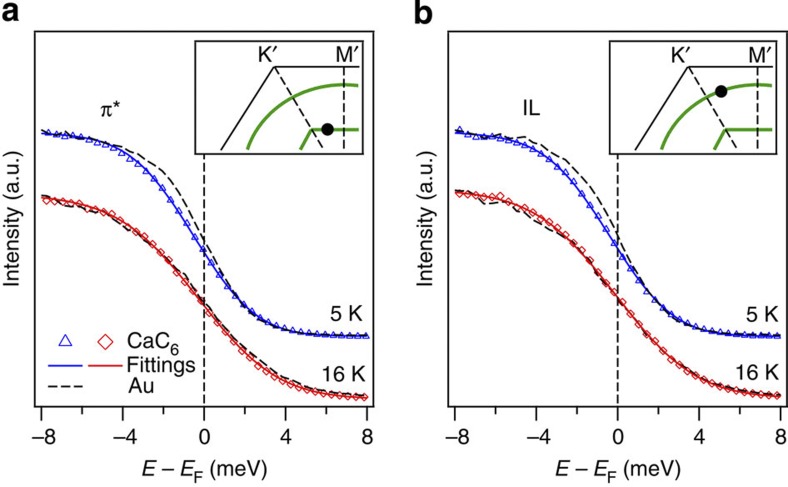
Opening of superconducting gaps. (**a**) Constant-momentum spectra acquired on the hexagonal π* band, both below (blue triangles) and above (red diamonds) *T*_c_. The spectra acquired on polycrystalline gold (dashed curves) are used to determine *E*_F_. All spectra are normalized by the values at 10 meV below *E*_F_ to account for the difference in photon flux and photoemission matrix element. The CaC_6_ spectra are fitted using a phenomenological model (solid curves)^21^. The model is built on a Fermi–Dirac distribution, multiplied by a linear function and convolved with the Gaussian resolution function. The inset shows the momentum for the spectral analysis. Owing to substantial resolution broadening (Methods), the leading edge shifts below *T*_c_ are significant underestimates of the actual gap values^18^,^19^,^20^, and are only used to indicate the opening of the gaps. (**b**) Spectra acquired on the IL band and reference gold. Notations and analysis are the same as those for **a**.

We identify the existence of superconducting gaps on the hexagonal π* and IL bands in Fig. 2a and b, respectively. Below the superconducting transition temperature (*T*_c_=11.5 K), the leading edges of the CaC_6_ spectra for both the π* and IL bands are clearly shifted to higher binding energies with respect to the spectra acquired on polycrystalline gold. Above *T*_c_, the leading edges of the CaC_6_ and gold spectra coincide. We also show in Supplementary Fig. 2 that the leading edge shifts below *T*_c_ are not confined to particular momenta. As demonstrated by previous ARPES experiments, leading edge shifts (Δ*E*) below *T*_c_ signal the existence of superconducting gaps^12^,^18^,^19^,^20^. By fitting the CaC_6_ spectra to a phenomenological model based on the Fermi–Dirac distribution^21^, we obtain Δ*E*_π*_=0.5±0.1 meV and Δ*E*_IL_=0.4±0.1 meV. We emphasize that our energy resolution contributes significant broadening (Methods). In this situation, leading edge shifts have been known to substantially underestimate the actual gap values^18^,^19^,^20^, and are not suitable for quantitative comparison to theory^5^ or other experiments^9^,^10^. Nevertheless, our analysis of the leading edge shifts clearly demonstrates the opening of superconducting gaps on both the π* and IL bands below *T*_c_.

Importantly, our leading edge measurement provides the first experimental evidence with clear momentum-space resolution for the existence of a superconducting gap on the π* band of any graphitic material. Tunnelling experiments have measured the superconducting gap in CaC_6_ and yielded a fully gapped picture, which is consistent with our results, but they were unable to directly resolve the separate contributions from the individual bands in momentum-space^9^,^10^. On the other hand, the ARPES study by Sugawara *et al.*^12^ attributed their measured gap near Γ to the IL band alone and reported no gap on the π* band. By resolving the distinct contributions from both bands, our measurement corrects this oversimplified assignment, and establishes the critical participation of both bands in superconductivity.

### EPC

Connecting to the debate on CaC_6_’s superconducting mechanism, our data resolve the controversy about which bands are superconducting, and agrees well with theory in this respect^5^. We now seek evidence for the phonon modes, which facilitate the superconducting transition^1^,^4^,^5^,^6^. The intense debate in the literature focused on whether the main contribution comes from the carbon in-plane mode (C_xy_)^13^, which couples only to the π* electrons, or the carbon out-of-plane (C_z_) and calcium in-plane (Ca_xy_) modes^1^,^4^,^5^,^6^, which mediate interactions between the graphene sheets and intercalant atoms. The key to revealing the superconducting mechanism is to separately characterize these different phonon modes and compare their respective EPC strengths.

**Figure 3:**
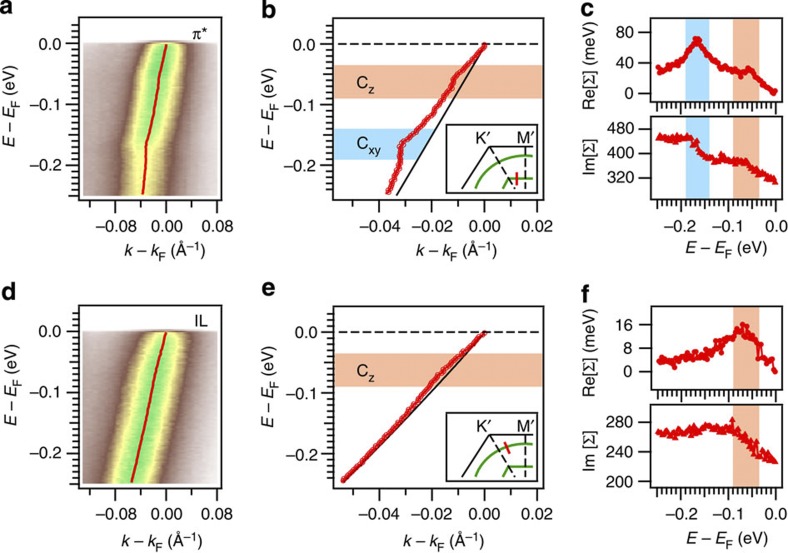
EPC in CaC_6_ at 5 K. (**a**,**d**) Typical ARPES cuts for the π* and IL bands, respectively. These cuts are taken at the same momenta as for the leading edge measurement in Fig. 2. The extracted band dispersions are overlaid on the raw data. (**b**,**e**) Identification of electron–phonon modes in the dispersions. The insets illustrate the momenta for the kink analysis. (**c**,**f**) Extracted real and imaginary parts of the electronic self-energies of the π* (**c**) and IL (**f**) bands.

In ARPES experiments, EPC is manifested in sudden changes in the electroic band dispersions and linewidths^11^,^22^,^23^,^24^, which, respectively, correspond to discontinuous features in the real and imaginary parts of the electronic self-energy (Re[Σ], Im[Σ]). In Fig. 3a,d, we present the ARPES cuts on the same momenta as for the leading edge measurement in Fig. 2. The momentum-distribution curves are analysed by fitting to Lorentzian distributions to extract the band dispersions and linewidths. The π* dispersion, shown in Fig. 3b, displays two kinks which are, respectively, near 160 meV and 50 meV below the Fermi level (*E*_F_). In contrast, Fig. 3e shows that the IL dispersion has only one pronounced kink near 70 meV. These kinks appear for all momenta along the respective Fermi surfaces (Supplementary Fig. 3). Based on an experimental phonon spectrum^25^ and theoretical calculations^1^,^4^,^5^,^6^, we assign the 160-meV kink to the coupling with the C_xy_ modes which span the range of 150–200 meV, and assign the 50 and 70 meV kinks to the coupling with the C_z_ modes which span 30–80 meV. We do not resolve any coupling with the Ca_xy_ modes ∼10 meV^4^, yet its existence cannot be ruled out owing to technical limitations such as the finite signal-to-noise ratio. Notably these results agree with theory^1^,^4^,^5^,^6^ on the existence of the C_z_ modes on both the IL and π* bands, along with the non-existence of the C_xy_ modes on the IL band. In Fig. 3c,f we extract the electronic self-energies on the π* and IL bands using the method described in the Methods section. At the corresponding mode energies, the peak features in Re[Σ] are one-to-one matched to the step features in Im[Σ], which corroborates our observation of the kinks in the band dispersions. Notably, both the Re[Σ] and Im[Σ] features have finite widths. The overlap in energy between the C_z_ features on the π* and IL bands indicates that there are common C_z_ modes which couple to both bands (Supplementary Note 1).

Importantly, our observation of the C_z_ modes on both the π* and IL bands evidences the interaction between the two bands^1^,^4^,^6^. To understand this point, consider the electron–phonon perturbation operator corresponding to the C_z_ modes. This operator has odd parity with respect to the *z* axis^6^, and can therefore only couple electronic states with opposite z-parities. This implies that intraband coupling to the C_z_ modes within the π* band is symmetry-forbidden, as the π* band has a universally odd z-parity^1^,^4^,^6^. On the other hand, the IL band has a strong contribution from the calcium 4*s* and 
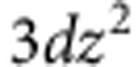
 orbitals^5^, both of which have even z-parities. Therefore, coupling to the C_z_ modes on the π* band is enabled by the existence of both bands^1^,^4^,^6^. The observation of the C_z_ modes in both bands provides direct evidence for this interband interaction.

**Figure 4:**
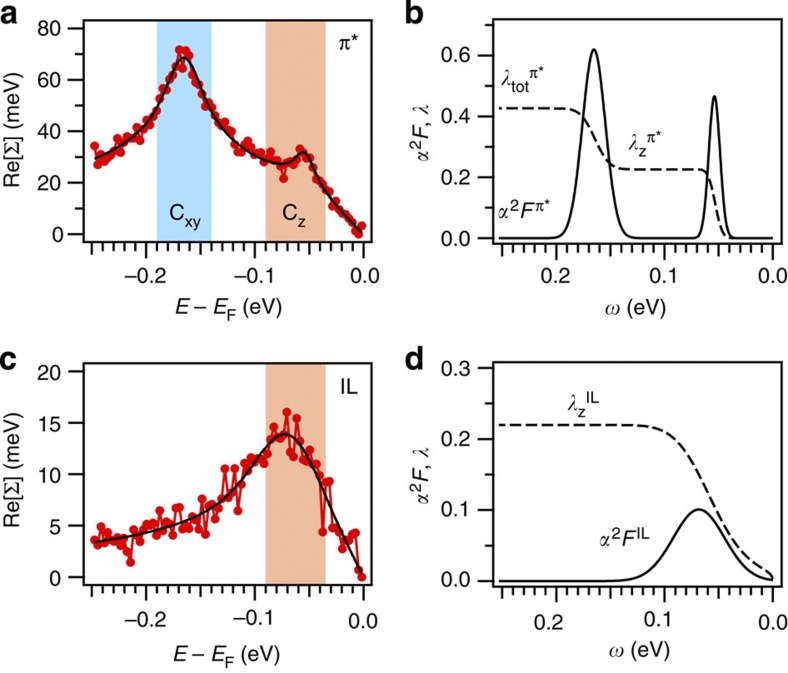
Evaluation of EPC strengths in CaC_6_. (**a**,**c**) Extracted real parts of the self-energies (solid circles) on the π* (**a**) and IL (**c**) bands based on the Eliashberg theory (Methods)^11^,^26^,^27^. (**b**,**d**) Extracted Eliashberg functions (α^2^*F*, solid) and EPC functions (*λ*, dashed) of the π* (**b**) and IL (**d**) bands. Here *ω* stands for the phonon frequency. The contributions to the total electron–phonon-coupling strengths are deduced: 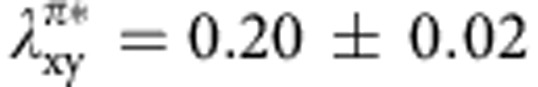, 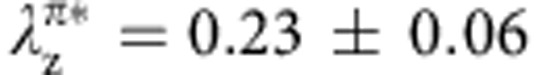, and 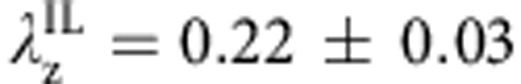.

We evaluate the EPC strengths (*λ*) to demonstrate the significant role of the interband interaction in superconductivity. Figure 4 presents the extracted Eliashberg functions, which can be subsequently used to deduce 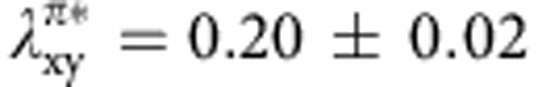, 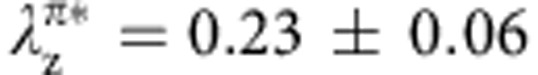 and 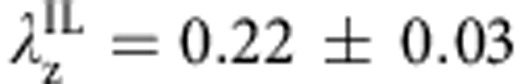 based on the Eliashberg theory as explained in the Methods section^26^,^27^. These values demonstrate that without the existence of the IL band and its associated interband interaction, the EPC strength on the π* band would be only 0.20, which is insufficient to facilitate the superconducting transition at 11.5 K (ref. 4). The contribution from the C_z_-mediated interband interaction to the total EPC strength on the π* band is >50%, which is crucial to enable superconductivity^1^,^4^,^6^. The same analysis performed at different momenta along the π* and IL bands also supports this conclusion (Supplementary Fig. 4).

## Discussion

Our analysis of the superconducting gaps and EPC strengths reveals that superconductivity of CaC_6_ cannot be attributed to either the π* or IL band alone. Instead, our discoveries demonstrate the critical role of the interaction between the two bands.

An important implication of this understanding is that the substantial contribution to the total EPC strength from the interband interaction can be readily explored in monolayer graphene. In this case, the IL band can be created by forming an array of surface adatoms with the resulting interband interaction then enabling superconductivity. This idea was recently utilized by Profeta *et al.*^1^ to predict that the monolayer LiC_6_ system may superconduct up to *T*_c_ ∼8.1 K. If this material can be fabricated, it will allow an efficient integration of various nanoscale technologies, such as superconducting quantum interference devices and single-electron superconductor-quantum dot devices^1^. Importantly, in this proposal the IL pocket size and the EPC strength contributed by the interband interaction are the keys to realizing the superconducting phase transition. Our work demonstrates the significant role ARPES will have in the implementation of this proposal, as it allows for direct measurement of not only the size of the IL band, but also the strength of the interband electron–phonon interaction. These results set a solid foundation for future exploratory activities in the pursuit of fabricating superconducting graphene devices.

### 

#### Note added in proof:

After the acceptance of our paper, we became aware of the ARPES study by Fedorov *et al.*^29^ which reports Ca-enhanced EPC on doped monolayer graphene.

## Methods

### Sample synthesis

High-quality CaC_6_ samples were synthesized from 3–4-mm platelets of natural single-crystal graphite using the lithium-alloy method^28^. The phase purity of the crystals was confirmed by X-ray diffraction to be >99% with no observable peaks from LiC_6_ or graphite impurities. A sharp superconducting transition was observed at 11.5 K using a SQUID magnetometer.

### ARPES measurement

Our ARPES measurement was performed at Beamline 5-4 of Stanford Synchrotron Radiation Light source using the Scienta R4000 electron analyser. Atomically clean surfaces were obtained by *in situ* cleaving in an ultra-high vacuum chamber with a pressure ∼2 × 10^−11^ Torr. The synchrotron light was incident on the sample surface with a linear polarisation perpendicular to the analyser slit in the π configuration. The measurement temperature was regulated to be stationary within 0.2 K. The energy resolution combining the broadenings from the radiation bandwidth and the analyzer slit was 5 meV. The angular (momentum) resolution was 0.2° (0.004 Å^−1^). Our results were repeated on multiple samples.

### Self-energy analysis

The connection between the Eliashberg function (*α*^2^*F*) and the real part of electron self-energy (Re[Σ]) is established in equation (1)^26^,^27^.


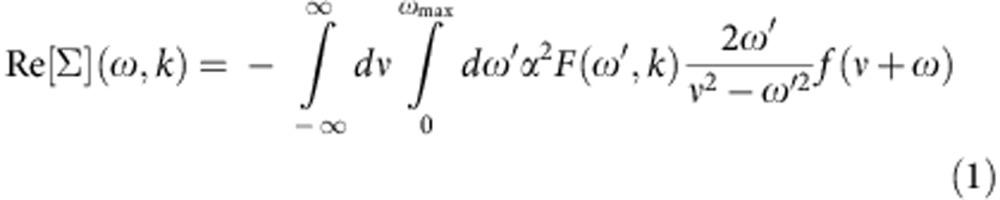


Here *f* is the Fermi–Dirac (FD) distribution; *ω*_max_ represents the maximum phonon frequency. At the low-temperature limit, the FD distribution becomes a step function, leading to a simplified formula as equation (2).


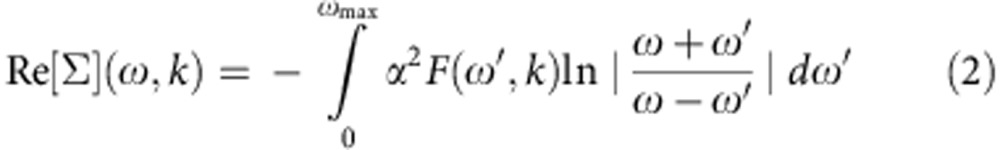


There is a well-established relation between Re[Σ] and the measured dispersion *ω*(*k*) from the ARPES literature^22^,^23^,^27^.


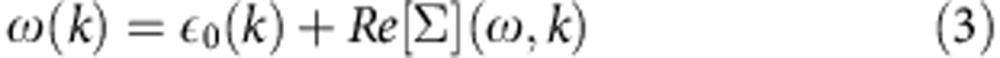


In principle, equations (2) and (3) allow us to extract α^2^*F* from a measured dispersion, yet the correct bare-band dispersion *ε*_0_(*k*) is *a priori* unknown. Conventionally, an ansatz is chosen for *ε*_0_(*k*), which is subtracted from *ω*(*k*) to yield Re[Σ]^11^,^22^. The shortcoming of this approach is that any subsequent analysis on Re[Σ] is highly sensitive to the original choice of *ε*_0_(*k*). Here we overcome this problem by fitting *ω*(*k*) in a single step, allowing the parameters describing both *ε*_0_(*k*) and Re[Σ] to vary freely. We find that all parameters are well constrained by the shape of *ω*(*k*), thus leading to a reliable extraction of both *ε*_0_(*k*) and Re[Σ]. This is demonstrated in Supplementary Fig. 5, where we use a linear function to parametrize *ε*_0_(*k*) for a π* dispersion^11^, and assume two Gaussian peaks in α^2^*F* to account for the C_xy_ and C_z_ modes. We find a drastic degradation in the fitting quality when we force the bare-band velocity to deviate 10% from the value determined by a free fit.

To fit IL dispersions, we use the ansatz of a single Gaussian peak for *α*^2^*F*. We also find that a parabolic bare-band dispersion is necessary for high-quality fitting, which is consistent with the free-electron-like nature of the IL band^1^,^2^,^3^,^4^,^5^,^6^.

The fitting procedure determines an effective *α*^2^*F*. The EPC function *λ*(*ω*,*k*) is subsequently deduced according to equation (4)^4^,^6^,^26^,^27^.


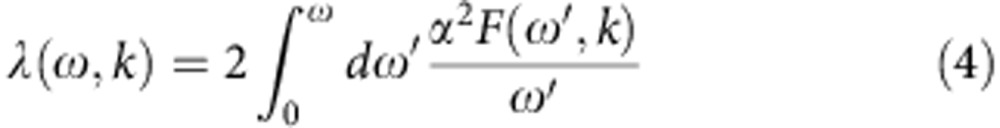


In Fig. 4, we illustrate how to obtain the respective coupling strengths. For the IL band, we integrate over the whole range (0–250 meV) to obtain 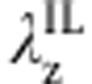. For the π* band, we selectively obtain 
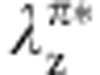
 or 
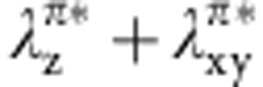
 by varying the integration window.

To estimate the uncertainties in the extracted EPC strengths, we consider in the *α*^2^*F* function a spectral peak represented by 
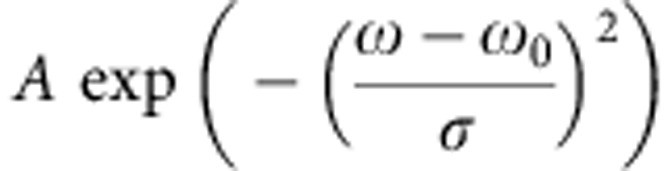
. Here *A*, *ω*_0_ and *σ* stand for the amplitude, the central energy and the peak width, respectively. If *ω*_0_≫*σ*, we can obtain an estimate of the EPC strength: 
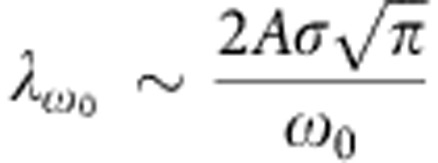
. According to the principle of error propagation, we deduce the uncertainty of 
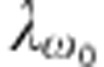
 using equation (5).


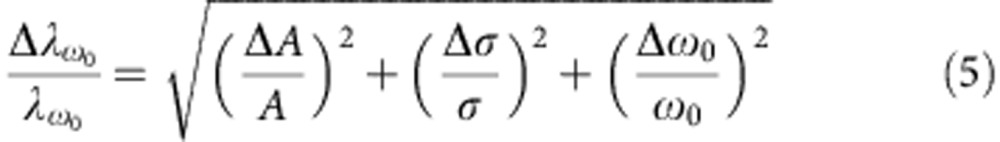



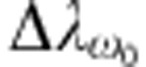
 is well determined as the quantities of the right-hand side of equation (5) can be all obtained from fitting the measured band dispersion.

## Author contributions

S.-L.Y. and J.A.S. carried out the experiment with support from M.H., D.H.L. and S.-K.M.; S.-L.Y. and J.A.S. analysed the data; S.-L.Y., J.A.S. and Z.-X.S. designed and coordinated the experiment; C.A.H. fabricated the samples; C.J.P. provided the theoretical input; S.-L.Y., J.A.S., P.S. K. and C.A.H. wrote the paper; all authors commented on the manuscript.

## Additional information

**How to cite this article:** Yang, S.-L. *et al.* Superconducting graphene sheets in CaC_6_ enabled by phonon-mediated interband interactions. *Nat. Commun.* 5:3493 doi: 10.1038/ncomms4493 (2014).

## Supplementary Material

Supplementary Information Supplementary Figures 1-5, Supplementary Note 1 and Supplementary References

## References

[b1] Profeta G., Calandra M. & Mauri F. Phonon-mediated superconductivity in graphene by lithium deposition. Nat. Phys. 8, 131–134 (2012).

[b2] Csányi G., Littlewood P. B., Nevidomskyy A. H., Pickard C. J. & Simons B. D. The role of the interlayer state in the electronic structure of superconducting graphite intercalated compounds. Nat. Phys. 1, 42–45 (2005).

[b3] Mazin I. I. Intercalant-driven superconductivity in YbC_6_ and CaC_6_. Phys. Rev. Lett. 95, 227001 (2005). 16384254 10.1103/PhysRevLett.95.227001

[b4] Calandra M. & Mauri F. Theoretical explanation of superconductivity in C_6_Ca. Phys. Rev. Lett. 95, 237002 (2005). 16384331 10.1103/PhysRevLett.95.237002

[b5] Sanna A. *et al.* Anisotropic gap of superconducting CaC_6_: a first-principles density functional calculation. Phys. Rev. B 75, 020511(R) (2007).

[b6] Boeri L., Bachelet G. B., Giantomassi M. & Andersen O. K. Electron-phonon interaction in graphite intercalation compounds. Phys. Rev. B 76, 064510 (2007).

[b7] Kim J. S., Kremer R. K., Boeri L. & Razavi F. S. Specific heat of the Ca-intercalated graphite superconductor CaC_6_. Phys. Rev. Lett. 96, 217002 (2006). 16803268 10.1103/PhysRevLett.96.217002

[b8] Lamura G. *et al.* Experimental evidence of s-wave superconductivity in bulk CaC_6_. Phys. Rev. Lett. 96, 107008 (2006). 16605783 10.1103/PhysRevLett.96.107008

[b9] Bergeal N. *et al.* Scanning tunneling spectroscopy on the novel superconductor CaC_6_. Phys. Rev. Lett. 97, 077003 (2006). 17026267 10.1103/PhysRevLett.97.077003

[b10] Gonnelli R. S. *et al.* Evidence for gap anisotropy in CaC6 from directional point-contact spectroscopy. Phys. Rev. Lett. 100, 207004 (2008). 18518573 10.1103/PhysRevLett.100.207004

[b11] Valla T. *et al.* Anisotropic electron-phonon coupling and dynamical nesting on the graphene sheets in superconducting CaC_6_ using angle-resolved photoemission spectroscopy. Phys. Rev. Lett. 102, 107007 (2009). 19392151 10.1103/PhysRevLett.102.107007

[b12] Sugawara K., Sato T. & Takahashi T. Fermi-surface-dependent superconducting gap in C_6_Ca. Nat. Phys. 5, 40–43 (2009).

[b13] Pan Z.-H. *et al.* Electronic structure of superconducting KC_8_ and nonsuperconducting LiC_6_ graphite intercalation compounds: evidence for a graphene-sheet-driven superconducting state. Phys. Rev. Lett. 106, 187002 (2011). 21635120 10.1103/PhysRevLett.106.187002

[b14] Calandra M., Attaccalite C., Profeta G. & Mauri F. Comment on ‘Electronic structure of superconducting KC_8_ and nonsuperconducting LiC_6_ graphite intercalation compounds: evidence for a graphene-sheet-driven superconducting state’. Phys. Rev. Lett. 108, 149701 (2012). 22540827 10.1103/PhysRevLett.108.149701

[b15] Pan Z.-H., Fedorov A. V., Howard C. A., Ellerby M. & Valla T. Reply to comment on ‘Electronic structure of superconducting KC_8_ and nonsuperconducting LiC_6_ graphite intercalation compounds: evidence for a graphene-sheet-driven superconducting state’. Phys. Rev. Lett. 108, 149702 (2012). 10.1103/PhysRevLett.106.187002 21635120

[b16] Calandra M. & Mauri F. Electronic structure of heavily doped graphene: the role of foreign atom states. Phys. Rev. B 76, 161406(R) (2007).

[b17] Kanetani K. *et al.* Ca intercalated bilayer graphene as a thinnest limit of superconducting C_6_Ca. Proc. Natl Acad. Sci. USA 109, 19610–19613 (2012). 23139407 10.1073/pnas.1208889109 PMC3511705

[b18] Loeser A. G. *et al.* Temperature and doping dependence of the Bi-Sr-Ca-Cu-O electronic structure and fluctuation effects. Phys. Rev. B 56, 14185–14189 (1997).

[b19] Zhang Y. *et al.* Nodeless superconducting gap in A_*x*_Fe_2_Se_2_ (A=K,Cs) revealed by angle-resolved photoemission spectroscopy. Nat. Mater. 10, 273–277 (2011). 21358648 10.1038/nmat2981

[b20] Zhang W. Photoemission Spectroscopy on High Temperature Superconductor: A Study of Bi_2_Sr_2_CaCu_2_O_8_ by Laser-Based Angle-Resolved Photoemission Springer (2013).

[b21] Armitage N. P. *et al.* A photoemission investigation of the superconducting gap in an electron-doped cuprate superconductor. J. Electron Spectrosc. Relat. Phenom. 114–116, 623–627 (2001).

[b22] Valla T., Fedorov A. V., Johnson P. D. & Hulbert S. L. Many-body effects in angle-resolved photoemission: quasiparticle energy and lifetime of a Mo(110) surface state. Phys. Rev. Lett. 83, 2085–2088 (1999).

[b23] LaShell S., Jensen E. & Balasubramanian T. Nonquasiparticle structure in the photoemission spectra from the Be(0001) surface and determination of the electron self energy. Phys. Rev. B 61, 2371–2374 (2000).

[b24] Vishik I. M. *et al.* Doping-dependent nodal Fermi velocity of the high-temperature superconductor Bi_2_Sr_2_CaCu_2_O_8+δ_ revealed using high-resolution angle-resolved photoemission spectroscopy. Phys. Rev. Lett. 104, 207002 (2010). 20867053 10.1103/PhysRevLett.104.207002

[b25] Dean M. P. M. *et al.* Neutron scattering study of the high-energy graphitic phonons in superconducting CaC_6_. Phys. Rev. B 82, 014533 (2010).

[b26] Grimvall G. The Electron-Phonon Interaction in Metals North-Holland Pub. Co. (1981).

[b27] Hofmann P., Sklyadneva I. Y., Rienks E. D. L. & Chulkov E. V. Electron-phonon coupling at surfaces and interfaces. New J. Phys. 11, 125005 (2009).

[b28] Pruvost S., Hérold C., Hérold A. & Lagrange P. Co-intercalation into graphite of lithium and sodium with an alkaline earth metal. Carbon 42, 1825–1831 (2004).

[b29] Fedorov A. V. *et al.* Observation of a universal donor-dependent vibrational mode in graphene. Nat. Commun. 5, 3257 (2014). 24500121 10.1038/ncomms4257

