# Supplementary material for: Superconducting graphene sheets in CaC6 enabled by phonon-mediated interband interactions
Source: Nat Commun. 2014 Mar 20;5:3493. doi: 10.1038/ncomms4493 (PMC3973042; doi:10.1038/ncomms4493)
Supplement: Supplementary Information — Supplementary Figures 1-5, Supplementary Note 1 and Supplementary References [file ncomms4493-s1.pdf]

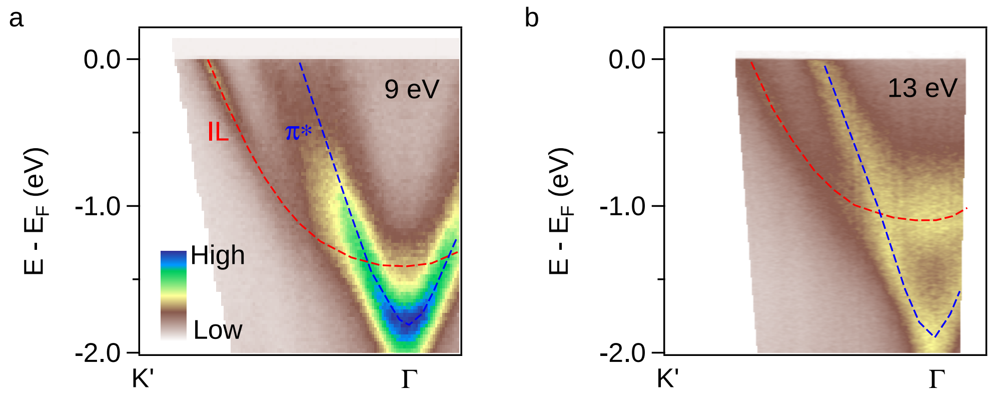

Supplementary Figure 1: ARPES cuts along Γ-K' using 9 eV (a) and 13 eV (b) photons. Red and blue guiding lines mark the IL and π^* dispersions, respectively. The 13 eV spectrum displays an upward shift of the IL band compared to that in the 9 eV spectrum. Since different photon energies probe different perpendicular momenta k_z [1,2], this shift demonstrates that the IL band disperses along k_z, and therefore has a three-dimensional electronic band structure. Considering that the IL band has a near-spherical Fermi surface (FS) [3–5], 9 eV photons indeed probe a k_z value closer to zero.

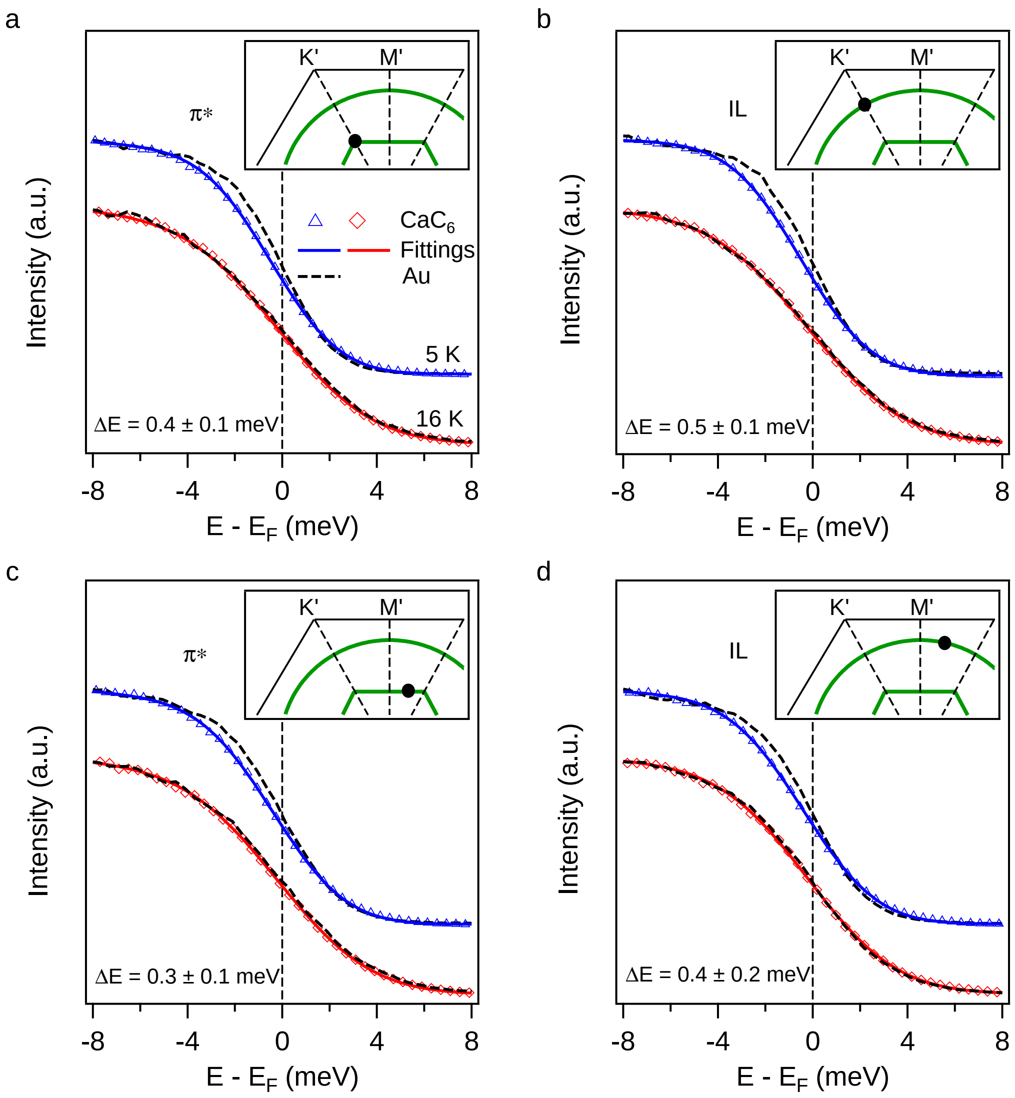

Supplementary Figure 2: Opening of superconducting gaps at various momenta on the π^* (a, c) and IL (b, d) bands. We adopt the same arrangement as in Fig. 2 of the main paper: the CaC_6 spectra below (blue triangles) and above (red diamonds) T_c are compared to the reference gold spectra (black dashed curves); fitting functions are plotted using solid curves. The insets illustrate the momenta for the corresponding measurements. The fitting results for the leading edge shifts are displayed in each panel. These results demonstrate the opening of non-zero superconducting gaps at various momenta.

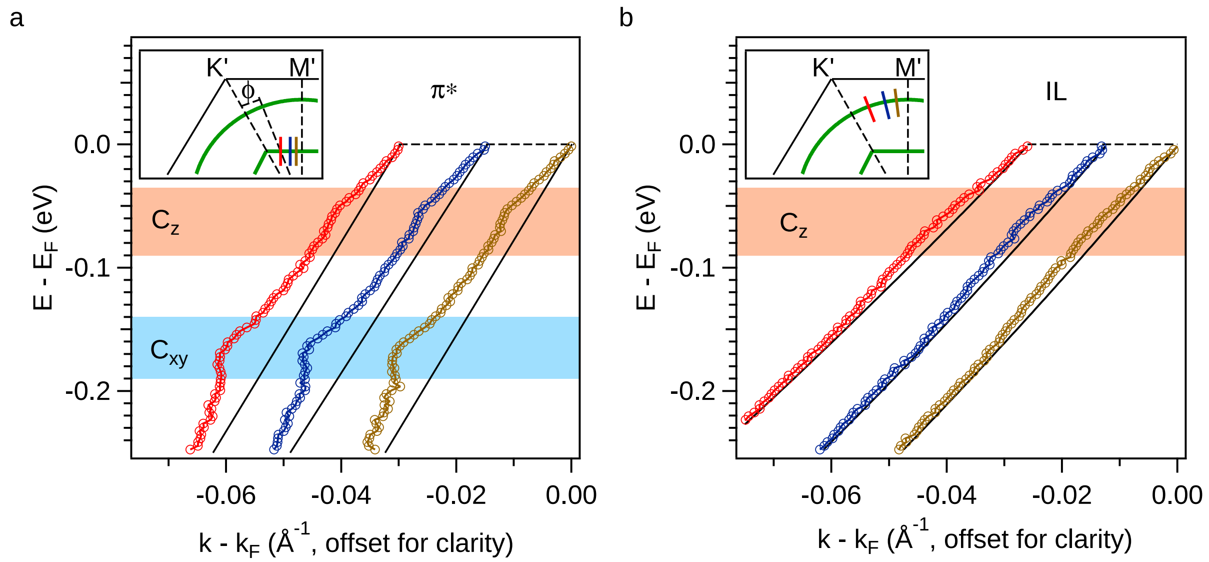

Supplementary Figure 3: Electron-phonon coupling at representative momenta at 5 K. (a) Extracted dispersions of the π^* band corresponding to $\phi = 9°$, $18°$, and $24°$ (from left to right) with respect to the Γ-K' direction. The inset illustrates the momenta for taking the ARPES cuts. The bare-band dispersions (black) are determined as discussed in the Methods section of the main text. (b) Dispersions of the IL band extracted at the same ϕ-values as in Panel (a). As seen in these band dispersions, the coupling with both the carbon in-plane (C_{xy}) and out-of-plane (C_z) modes exists on the π^* band, while the coupling with only the C_z modes exists on the IL band. Following the same reasoning as in the main text, these results suggest that the interband interaction occurs at all momenta.

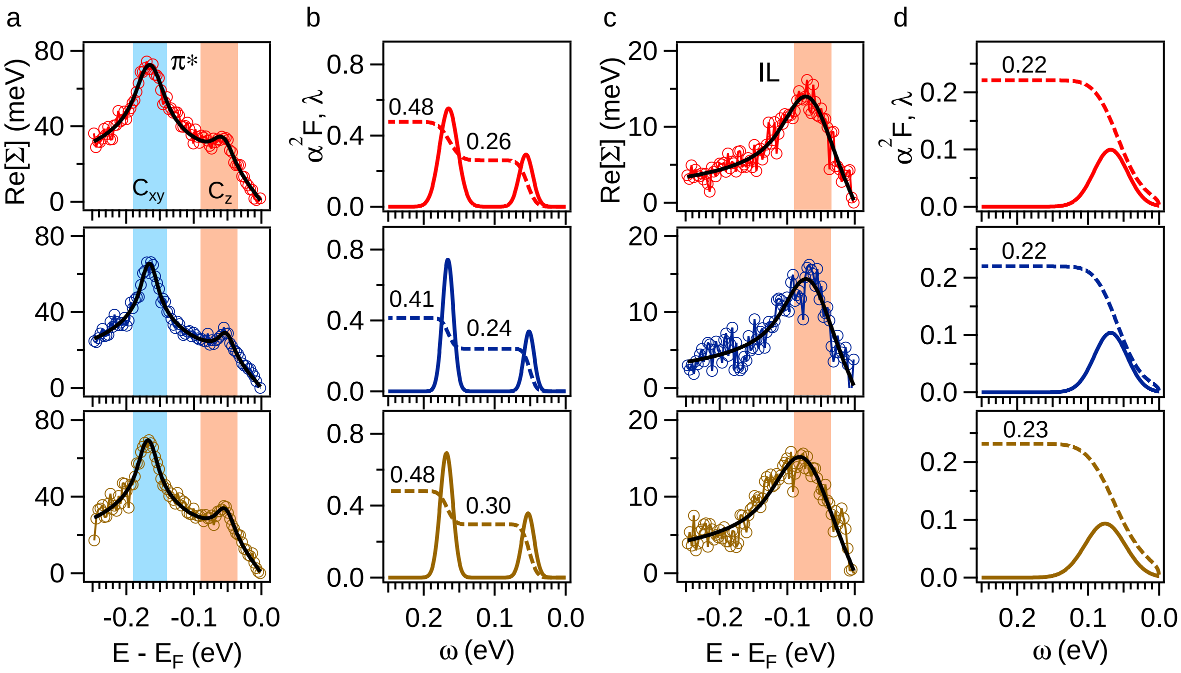

Supplementary Figure 4: Additional kink analysis at representative momenta. (a, c) Extracted real parts of the self energies (hollow circles) on the π^* (a) and IL (c) bands at $\phi = 9°$ (red), $18°$ (blue), and $24°$ (brown) as defined in Supplementary Fig. 3. The bare-band dispersions are described by free fitting parameters as discussed in the Methods section of the main text. (b, d) Extracted Eliashberg functions (α^2F, solid) and electron-phonon coupling functions (λ, dashed) of the π^* (b) and IL (d) bands. The C_z-associated and total coupling strengths are marked in (b) and (d). When comparing with theory[3], $\lambda_{xy}^{\pi*}$ (0.17∼0.22) falls in the theoretical range (0.1∼0.2), while $\lambda_z^{\pi*}$ (0.23∼0.3) and λ_z^{IL} (0.22∼0.23) do not agree with theory (0.4∼0.8). Note that this is not particularly surprising as there are intrinsic uncertainties in the calculations based on the density-functional theory (DFT) due to different approximations for exchange correlation functionals. For example, Sanna *et al.* reported a total electron-phonon coupling constant of 0.85,[3] whereas Profeta *et al.* reported 0.68 based on a different DFT implementation[5]. In this respect, our experimental results are important for further refinement of the DFT calculations.

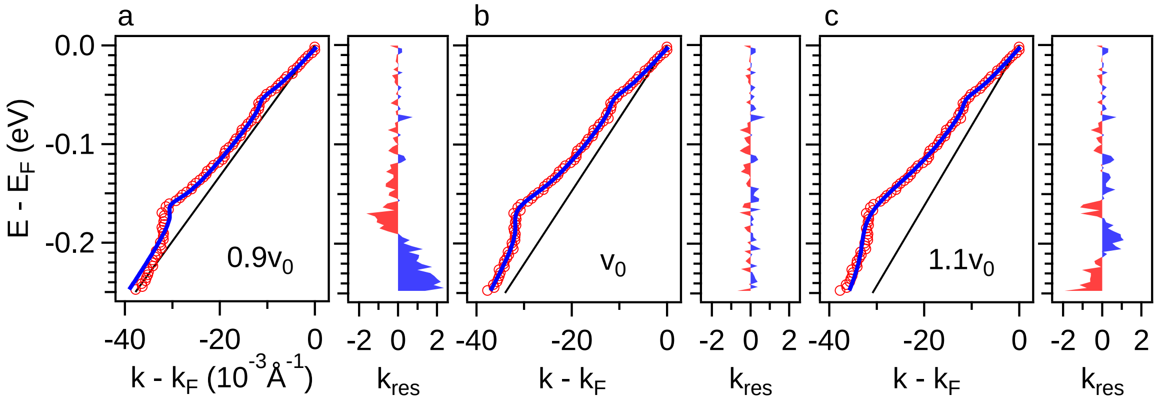

Supplementary Figure 5: Comparison of fitting results on the π^* band with different bare-band velocities. Panel (b) illustrates the optimal result when the bare-band velocity (v_0) is a free fitting parameter. From this fitting we obtain $v_0 = 7.35 \pm 0.04$ (eVÅ). Panels (a) and (c) display the results when the bare-band velocity is forced to be $0.9v_0$ and $1.1v_0$. In all panels, the experimental data and the fitting functions are plotted by red circles and blue curves, respectively. The bare-band dispersions are represented by black lines. The fitting residuals ($k_{\rm res}$) are displayed for comparison of the fitting qualities. The fitting is drastically degraded when we force the bare-band velocity to deviate from v_0.

Supplementary Note 1

The central energies of the C_z peaks are slightly different on the π^* and IL bands (Fig. 4 of the main text and Supplementary Fig. 4). Two things have to be considered to understand this difference. First, these spectral peaks have finite widths. The C_z peaks of the IL band span the range of 30$\sim$100 meV, and those of the π^* band span 40$\sim$60 meV. Therefore, the C_z modes which couple to the π^* band are well contained in the C_z peaks of the IL band, which supports the picture of interband interaction. The second issue to consider is that the C_z peaks of the IL band may include contributions other than the interband interaction. In particular, intraband interaction mediated by the C_z modes may take place on the IL band, because it has contributions from both even (Ca $4s$ and $3d_{z^2}$) and odd (C p_z) orbitals[4]. This

is consistent with our observation that the C_z peaks on the IL band are broader and contain the C_z peaks on the π^* band.

Supplementary References

1. Hüfner, S. *Photoelectron Spectroscopy: Principles and Applications.* (Springer, Berlin, 2003).

2. Chiang, T.-C., Knapp, J. A., Aono, M. & Eastman, D. E. Angle-resolved photoemission, valence-band dispersions $E(\mathbf{k})$, and electron and hole lifetimes for GaAs. *Phys. Rev. B* **21,** 3513–3522 (1980).

3. Sanna, A. *et al.* Anisotropic gap of superconducting CaC_6: a first-principles density functional calculation. *Phys. Rev. B* **75,** 020511(R) (2007).

4. Mazin, I. I. *et al.* Unresolved problems in superconductivity of CaC_6. *Physica C* **460–462,** 116–120 (2007).

5. Profeta, G., Calandra, M. & Mauri, F. Phonon-mediated superconductivity in graphene by lithium deposition. *Nature Phys.* **8,** 131–134 (2012).
